# Characterization of HIV-1 diversity in various compartments at the time of primary infection by ultradeep sequencing

**DOI:** 10.1038/s41598-020-59234-6

**Published:** 2020-02-12

**Authors:** Géraldine Gaube, Alix Armero, Maud Salmona, Marie-Laure Néré, Nadia Mahjoub, Caroline Lascoux-Combe, Audrey Gabassi, Sébastien Gallien, Ali Amara, Jean Michel Molina, Constance Delaugerre, Marie-Laure Chaix

**Affiliations:** 1 AP-HP, Hôpital Henri Mondor, Service d’Immunologie et Maladies Infectieuses, Université Paris Est Créteil, Inserm U955, Créteil, France; 2 AP-HP, Hôpital Saint-Louis, Virologie, Paris, France; 3 INSERM UMR 976, Université de Paris, Paris, France; 4 CNR VIH, Paris, France; 5 AP-HP, Hôpital Saint Louis, SMIT, Paris, France; 6 INSERM UMR 944, Université de Paris, Paris, France

**Keywords:** Evolution, Microbiology, Molecular biology

## Abstract

We used next-generation sequencing to evaluate the quantity and genetic diversity of the HIV *envelope* gene in various compartments in eight patients with acute infection. Plasma (PL) and seminal fluid (SF) were available for all patients, whole blood (WB) for seven, non-spermatozoid cells (NSC) for four, and saliva (SAL) for three. Median HIV-1 RNA was 6.2 log_10_ copies/mL [IQR: 5.5–6.95] in PL, 4.9 log_10_ copies/mL [IQR: 4.25–5.29] in SF, and 4.9 log_10_ copies/mL [IQR: 4.46–5.09] in SAL. Median HIV-1 DNA was 4.1 log_10_ copies/10^6^ PBMCs [IQR: 3.15–4.15] in WB and 2.6 log_10_ copies /10^6^ Cells [IQR: 2.23–2.75] in NSC. The median overall diversity per patient varied from 0.0005 to 0.0232, suggesting very low diversity, confirmed by the clonal aspect of most of the phylogenetic trees. One single haplotype was present in all compartments for five patients in the earliest stage of infection. Evidence of higher diversity was established for two patients in PL and WB, suggesting compartmentalization. Our study shows low diversity of the *env* gene in the first stages of infection followed by the rapid establishment of cellular reservoirs of the virus. Such clonality could be exploited in the search for early patient-specific therapeutic solutions.

## Introduction

Understanding the dynamics of human immunodeficiency virus type 1 (HIV-1) transmission is important in the design of effective prevention and treatment strategies. Several studies suggest that early stages of HIV infection may disproportionately contribute to viral transmission and spread of the epidemic^1^. Indeed, recent infection, particularly primary HIV infection (PHI), is associated with a high viral burden in blood and semen, a major determinant of HIV transmission^2–5^. Within the first weeks of infection, HIV rapidly disseminates throughout the body and establishes cellular HIV reservoirs and compartments^6^. Phylogenetic analyses of founder viruses in various epidemic settings support the notion of a genetic bottleneck, with only a single founder in almost all cases of sexual transmission^7,8^. Such a genetic bottleneck leads to low genetic diversity and clonal representation of the viral population in patients with a PHI. Several biological factors have been suggested to be responsible, including the mucosa in the sexual tract^9^, the availability of target cells^10^, and the levels of immune activation and genital inflammation^11^.

Viral compartmentalization within anatomical regions has been documented in PHI, mainly in the central nervous system and genital tract^6^. This is a consequence of restricted viral migration between anatomical sites or tissues^12^. Such compartmentalization affects HIV-associated pathogenesis and is involved in neurocognitive disease^13^ and sexual transmission^14–16^. For example, the male genital tract represents an unique compartment, with differences in viral replication and specific evolution in response to local environmental factors^17–19^.

Here, we used ultra-deep sequencing (UDS) to determine the quantity and genetic diversity of the HIV *envelope* gene to assess the diversity of the virus and characterize the dynamics of viral spread between several compartments (plasma, whole blood, seminal fluid, non-spermatozoid cells, and saliva) in patients with a primary infection.

## Results

### Patient characteristics

Eight patients (P1 to P8) were enrolled in this study at the time of PHI. The clinical characteristics of each are described in Table 1. All were men with a median age of 37.5 years (seven reporting sex with men and one reporting heterosexual behavior). Primary infection was symptomatic in four cases. Median CD4 cell counts and HIV-1 RNA levels were 523 cells/mm^3^ (range: 103–707) and 6.2 log_10_ copies/mL [range: 5.5–6.95], respectively. One patient was classified as Fiebig II, two as Fiebig IV, and five as Fiebig V. Four patients have a negative HIV serological test in the last 3 months before the study. Using tool for HIV estimation date^20^, 7 out of the 8 patients, had an estimated date of infection lower than 30 days. Six patients were infected with a subtype B virus and two with CRF02_AG. Viral tropism was CCR5 in seven cases and CCR5/CXCR4 in one.

**Table 1:** Clinical, biological and behavioral characteristics of the patients.

	P1	P2	P3	P4	P5	P6	P7	P8
Age (years)	30	25	39	39	27	36	43	43
Sex	M	M	M	M	M	M	M	M
Country of birth	Guinea	Brazil	France	France	France	France	Peru	France
Transmission group	HTS	MSM	MSM	MSM	MSM	MSM	MSM	MSM
STI in the last 3 months	no	no	no	no	yes	no	no	no
PHI Symptoms	Fever, diarrhea	Abdominal pain, headache	Fever, rash, diarhea, pharyngitis	none	none	none	Fever, asthenia	unknown
Fiebig stage	V	II	IV	V	V	V	V	IV
CD4 /mm^3^	103	484	542	504	547	707	437	588
CD4/CD8	0.89	1.1	0.6	0.35	0.42	0.59	0.5	0.76
HIV-RNA log_10_ copies/ml	7.49	6.26	6.78	6.06	4.38	4.59	5.80	7.46
HIV-1 subtype	CRF02_AG	B	B	B	B	B	CRF02_AG	B
Tropism	X4/R5	R5	R5	R5	R5	R5	R5	R5

P: patient, HTS: heterosexual, MSM: men who have sex with men, STI: sexual transmitted infection, PHI: primary infection.

### Quantification of HIV-1 RNA and DNA

**Figure 1:**
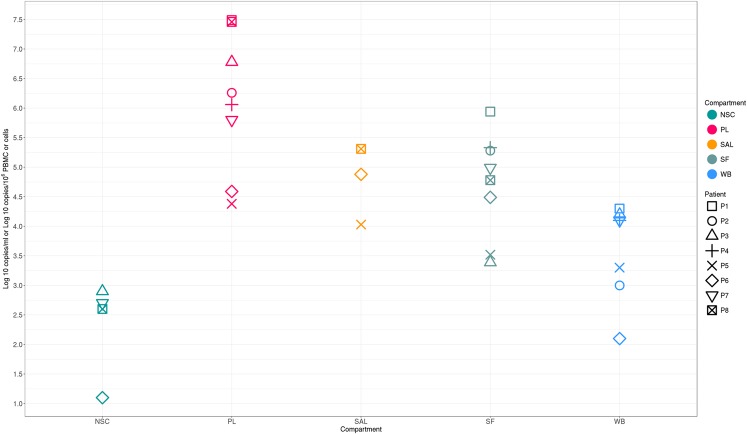
HIV viral loads in five compartments of patients with primary infection. HIV RNA was estimated in plasma (PL), saliva (SAL), and seminal fluid (SF) expressed in log_10_ copies/ml. HIV DNA was quantified in PBMCs (WB) and non-spermatozoid cells (NSC) and expressed in log_10_ copies/10^6^ PBMCs or cells, respectively. P: patient.

Plasma (PL) and seminal fluid (SF) were available for all eight patients, whole blood (WB) for seven, non-spermatozoid cells (NSC) for four, and saliva (SAL) for three. The median HIV-1 RNA level was 6.2 log_10_ copies/mL [IQR 5.5–6.95] in PL, 4.9 log_10_ copies/mL [4.25–5.29] in SF, and 4.9 log_10_ copies/mL [IQR 4.46–5.09] in SAL. The median HIV-1 DNA level was 4.1 log_10_ copies/10^6^ PBMC [IQR 3.15–4.15] in WB and 2.6 log_10_ copies /10^6^ cells [IQR 2.23–2.75] in NSC. During PHI, PL HIV RNA levels were higher than those in all other compartments for seven patients, whereas the viral load (VL) in SAL was slightly higher than that in PL (4.88 log_10_ vs 4.59 log_10_) for the remaining patient (P6)  (Fig. 1).

### Diversity of the *env* gene

**Figure 2:**
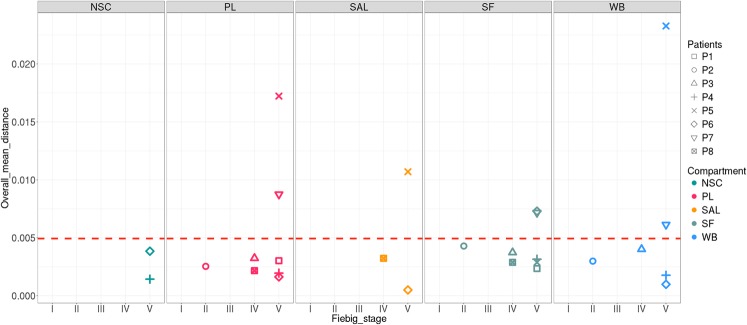
Average diversity of HIV sequences in compartments from patients with a primary infection according to Fiebig stage. The estimated overall mean distance of the reads is represented for each of the compartments. NSC: non-spermatozoid cells, PL: plasma, SAL: saliva, SF: seminal fluid, WB: whole blood.

We sequenced the C2V3 region between positions 7008–7385 bp of the HXB2 reference sequence using UDS. Amplification was performed for the eight SF samples and seven PL, six WB, three SAL, and two NSC samples. After read filtration based on quality parameters, we estimated a median of 5,792 representative sequences for each sample, with an average length of 200 bp and a deep average of 5,562 reads by position. The overall mean distance i.e. the mean pairwise genetic Tamura Nei^21^ distance between reads in each compartment is represented in Fig. 2.

Diversity estimates were very low, from 0.0005 to 0.0232. For most of the analyzed samples, the diversity was 0.005, with some exceptions, such as PL and WB for P5 and P7, SAL for P5, and SF for P6 and P7.

Figure 2 shows a tendency to increase the dispersion of mean diversity estimates among Fiebig stage V patients. To assess whether there is a differential pressure between the compartments in Fiebig stage IV and V patients, we evaluated the relationship between diversity and compartment and Fiebig stage with a generalized linear mixed models. These models didn’t find a significant association between the compartment and/or Fiebig stage with the diversity (Supplementary Note S1). The lack of effect of the compartments on the diversity of Fiebig stage IV and V patients can be observed in Supplementary Fig. S1.

### Haplotype and phylogenetic analysis

**Figure 3:**
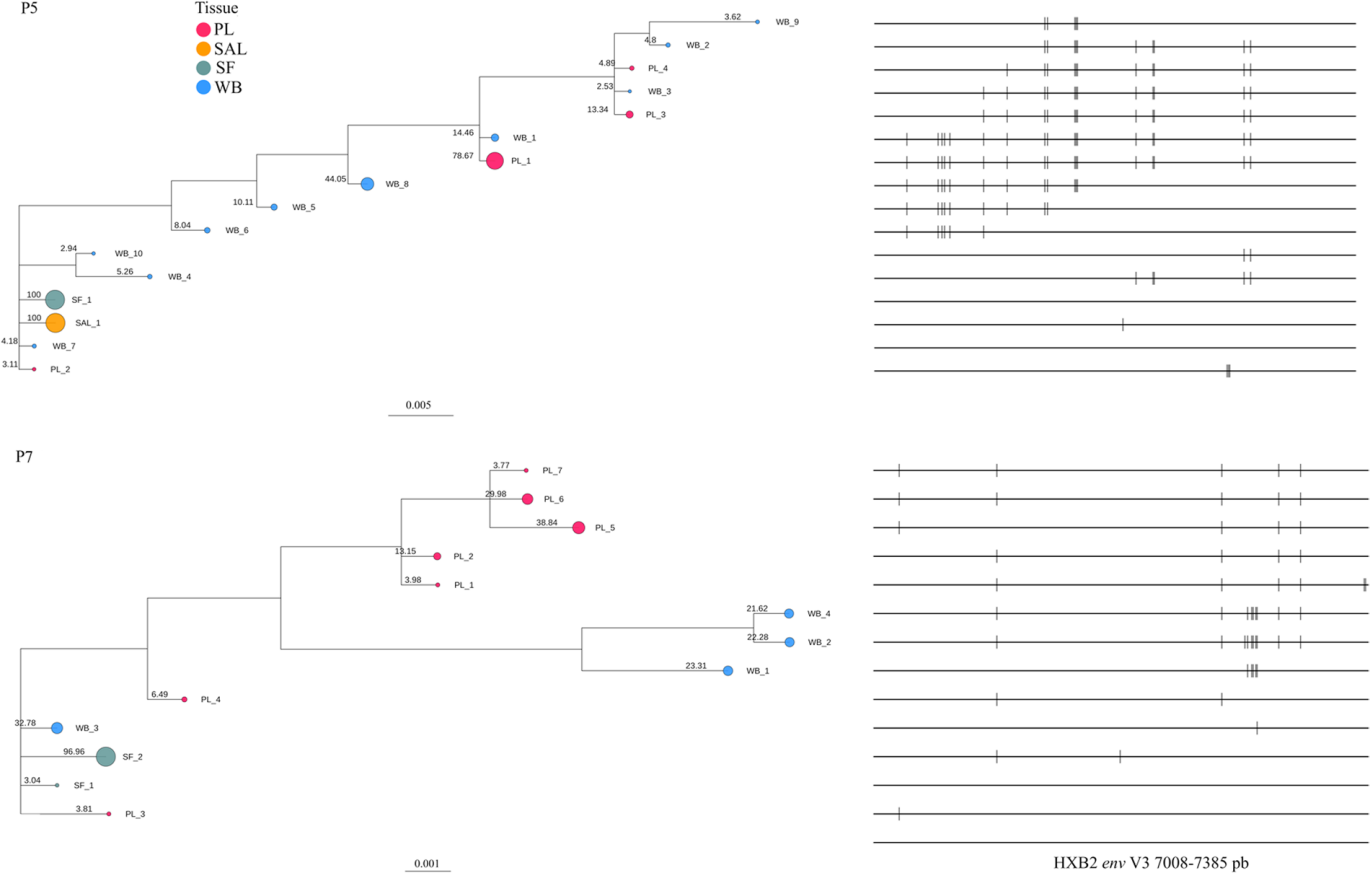
Phylogenetic trees of the HIV haplotypes identified in Patients 5 and 7. The size of the circumference in the phylogenetic trees is proportional to the frequency of the haplotype in the respective compartment and is indicated for each branch. The nucleotide substitutions identified by the Highlighter tool (www.hiv.lanl.gov) are represented at the front of each haplotype.

Haplotype analysis was performed for seven of the eight patients, as we could not recover viral haplotypes from P1 due to low sequence coverage (Supplementary table 1). The patients could be divided into two groups based on the number and diversity of the haplotypes. In the first (P2, P3, P4, P6, P8), each compartment of the same patient showed one or two haplotypes, with high sequence similarity (intra-patient). Phylogenetic trees confirmed low diversity for P2, P3, P4, P6, and P8, with a clonal aspect and the characteristic star-like phylogeny (Supplementary Figure 2). The second group of patients, consisting of P5 and P7, showed greater diversity, with 16 and 13 haplotypes, respectively. The compartment with the highest number of haplotypes in P7 was the PL (n = 7), whereas WB was the most diverse compartment for P5 (n = 10). The phylogenetic tree for P5 showed high diversity and a specific pattern of nucleotide variation, suggesting potential compartmentalization. Similarly, we found distinct compartment-specific clusters of variants in the blood and plasma of P7 (Fig. 3).

### Evidence of compartmentalization in later Fiebig stages

The results of the Fst and Slatkin-Maddison tests are presented in Table 2. We found no evidence of compartmentalization among the various compartments of P2, P3, P6, or P8. P4 showed NSC compartmentalization relative to the other compartments (WB, PL, SF), P5 compartmentalization among all compartments sampled (PL, WB, SAL, SF), and P7 significant divergence between the WB-PL and PL-SF pairs.

**Table 2:** Evaluation of the compartmentalization between pairs of samples from the same patient with Wright’s measure (Fst). The p-value was calculated by randomly assigning the sequences to the compartments and re-calculate the Fst. 100 resampling were performed in each pair of comparisons.

Patient Pairs of compartments		Fst all reads		Fst one sequence by Cluster		Slatkin-madisson	
		Fst estimated	P-value	Fst estimated	P-value	% of significant permutations	Comparmentalization
PHI2	WB-PL	0.018	0.314	0.001	0.412	13	No
PHI2	WB-SF	0.014	0.275	−0.001	0.667	38	No
PHI2	PL-SF	0.069	0.010	0.001	0.245	29	No
PHI3	WB-PL	−0.009	0.637	−0.046	0.931	1	No
PHI3	WB-SF	0.012	0.363	0.004	0.451	2	No
PHI3	PL-SF	−0.007	0.559	−0.021	0.618	1	No
PHI4	WB-NSC	0.693	<0.0001*	0.316	<0.0001*	100	Yes
PHI4	WB-PL	0.028	0.216	0.010	0.069	44	No
PHI4	PL-NSC	0.690	<0.0001*	0.304	<0.0001*	100	Yes
PHI4	WB-SF	0.223	<0.0001*	0.046	<0.0001*	58	No
PHI4	NSC-SF	0.692	<0.0001*	0.286	<0.0001*	100	Yes
PHI4	PL-SF	0.690	<0.0001*	0.018	<0.0001*	16	No
PHI5	PL-WB	0.273	<0.0001*	0.157	<0.0001*	100	Yes
PHI5	WB-SF	0.412	<0.0001*	0.196	<0.0001*	100	Yes
PHI5	PL-SF	0.892	<0.0001*	0.752	<0.0001*	100	Yes
PHI5	PL-SAL	0.653	<0.0001*	0.556	<0.0001*	100	Yes
PHI5	WB-SAL	0.119	<0.0001*	0.157	<0.0001*	100	Yes
PHI5	SF-SAL	0.735	<0.0001*	0.495	<0.0001*	100	Yes
PHI6	WB-NSC	0.029	0.225	0.073	0.382	8	No
PHI6	WB-PL	0.167	0.059	0.009	0.402	6	No
PHI6	NSC-PL	−0.260	0.853	−0.120	0.706	2	No
PHI6	WB-SF	0.390	0.049	0.117	0.098	11	No
PHI6	NSC-SF	0.145	0.147	−0.031	0.559	16	No
PHI6	PL-SF	−0.157	0.941	−0.064	0.657	13	No
PHI6	WB-SAL	−0.115	0.902	−0.066	0.931	4	No
PHI6	NSC-SAL	−0.234	0.902	−0.362	0.863	3	No
PHI6	PL-SAL	0.227	0.020	−0.066	0.922	8	No
PHI6	SF-SAL	0.169	0.137	−0.286	0.941	14	No
PHI7	PL-WB	0.208	<0.0001*	0.111	<0.0001*	100	Yes
PHI7	WB-SF	−0.002	0.529	−0.010	0.824	100	No
PHI7	PL-SF	0.284	<0.0001*	0.135	<0.0001*	100	Yes
PHI8	PL-SF	−0.041	0.873	−0.032	0.941	75	No
PHI8	PL-SAL	−0.019	0.725	−0.020	0.951	59	No
PHI8	SF-SAL	0.022	0.284	−0.002	0.716	44	No

*Significant p-values, SF: seminal fluid, WB: Whole blood, PL: Plasma, SAL: saliva, NSC: non spermatozoid cells.

### Role of positive selection in the compartmentalization of patients with primary infection

We evaluated the evidence of positive selection in the HIV haplotypes for each patient. Only P5 and P7 showed evidence of positive selection in the PL and WB compartments. Many of the amino-acid changes in the HIV haplotypes of P5 (10/13, 77%) and P7 (6/11, 55%) were under positive selection (Supplementary Fig. S3).

We performed a factorial correspondence analysis to establish whether these amino-acid changes under positive selection were a sign of a potential compartmentalization process. These substitutions were unable to discriminate the haplotypes depending on the compartment for P5. Conversely, the mutations under positive selection separated the haplotypes of the PL from WB compartment for P7. The three mutations with the strongest discriminant power for P7 were G358W, K359Q, and D268N. We investigated whether any mutations under positive selection could be a potential glycosylation site and identified only the D7N mutation (Supplementary Fig. S3).

## Discussion

At the best of our knowledge, this is the first study to analyze the quantity and genetic diversity of HIV in different compartments (blood, genital compartment, and saliva) in patients with a primary infection. HIV-RNA levels were high in semen (median 4.9 log_10_ copies/ml), albeit lower than in PL, consistent with the results of previous studies in PHI. The burden of the presence of HIV particles in semen can be particularly critical for the risk of transmission, especially in MSM^2,22–24^. We also found a high level of HIV RNA in SAL for the three patients with available samples. In a recent study, Ikeno *et al*. reported that the salivary viral load is approximately 10% of the PL viral load but that it can be even higher than the PL viral load in some patients^25^. In contrast to SF, SAL has been shown to lyse HIV particles *in vitro* due to hypotonicity and many salivary proteins inhibit and inactivate HIV particles^26^. The high and similar amounts of HIV RNA in the cell-free compartments suggest the passive diffusion of HIV from PL to the SF and SAL. The median level of HIV DNA in WB was 4.1 log_10_ copies/10^6^ PBMCs, suggesting the very early establishment of a cell reservoir, as previously described^2^. Conversely, we found low levels of HIV DNA in NSC, suggesting that the semen reservoir is established later than the blood reservoir during PHI.

Overall, we found little diversity in the HIV-1 quasispecies populations in compartments in eight men with acute infection. Our findings are compatible with a very early HIV-1 transmission bottleneck. The absence of structure of the phylogenetic trees and the small number of haplotypes favor single transmission for most patients. The percentage of sexual transmission events involving a single variety of HIV has been estimated to be from 76% to 80%^7,9^. Whether the percentage of patients with multiple variants could be greater among MSM patients is a subject of debate^27^. Our results do not support the multiple-transmission hypothesis, although we cannot exclude the possibility that the subjects were exposed to a relatively homogeneous viral population (if the transmitting partners had acute infections themselves).

The homogeneity of viral haplotypes suggests effective dispersion of the founder haplotype or a single haplotype derived early after transmission. The homology of the SF and PL haplotypes is evidence that the cell-free viral quasispecies in the genital compartment probably arose from PL. Such a flow could gradually create a cellular reservoir of the virus^2^, which could emerge in case of a break from antiretroviral treatment^28^. This may also be true for saliva based on our analysis. However, more studies are necessary to determine the presence of a viral reservoir in SAL.

We found evidence of compartmentalization in two of the patients, according to the compartmentalization tests and the phylogenetic analysis of the haplotypes. In these patients, WB and PL were the compartments that present the greatest diversity of haplotypes and reads. The haplotypes of these compartments provide evidence of positive selection probably as a response to the action of neutralizing antibodies^29^. Generalized linear mixed models didn’t identify differential pressure between the compartments of the patients in late Fiebig stage. This result could be due to the small number of patients.

The genetic homogeneity of the viral population in primary infection, independent of the compartment, has relevant implications for treatment of the disease. The latest proposed therapies aim to boost the response of the immune system using vectors such as DNA, recombinant virus, or dendritic cells^30^. Some have focused on the first stages of infection, such as the canarypox vaccine, without significant results^31^. However, approaches that simultaneously address the primary and secondary immune response, such as dendritic cells, could achieve better results.

Our study had several limitations. A longitudinal study is probably better adapted for the analysis of diversity and the evaluation of compartmentalization. Indeed, obtaining samples at various timepoints would allow a detailed analysis of the population dynamics within and between compartments. We also did not have homogeneous representation of patients in the different Fiebig stages and there were large differences in the number of samples available for the various compartments. Although these limitations may have introduced biases, we believe that more representative sampling would confirm our results.

UDS technique required quality correction before analyses; such correction may possibly affect the diversity. So, we compare our diversity data with previous work also using amplification of HIV *env* region (C2V3) with UDS and focusing on chronically infected patients^32,33^. These studies find higher diversity than our study on several compartments (blood, plasma semen and CSF) with a similar methodological approaches and quality correction. These data suggest that our methodological approach is able to identify high diversity in compartment.

In conclusion, we evaluated the genetic compartmentalization of the HIV population in plasma, whole blood, saliva, non-spermatic cells, and seminal fluid in patients with primary HIV infection. This study found a low C2V3 diversity in the first stages of infection and the rapid establishment of cellular reservoirs of the virus. Such clonality could be exploited in the search for early patient-specific therapeutic solutions.

## Methods

### Study population

Eight patients were diagnosed at the time PHI in the department of infectious diseases in Saint-Louis Hospital, Paris, between July and October 2017. PHI was confirmed by one of the following criteria: (i) positive 4^th^ generation HIV-1 ELISA with a negative or incomplete western blot (no anti-p68 or anti-p34) or (ii) positive p24-antigen ELISA and positive for HIV-1 RNA with a negative western blot. Patients were further categorized using adapted Fiebig (F) stages^34^ as follows: FI: HIV RNA^+^, p24^−^, antibody EIA^−^, WB^−^; FII: HIV RNA^+^, p24^+^, antibody EIA^−^, WB^−^; FIII: HIV RNA^+^, p24^+^, antibody EIA^+^, WB^−^; FIV: HIV RNA^+^, p24^+/−^, antibody EIA^+^, indeterminate WB (<3 antibodies or <2 specific antibodies from among gp160, gp120, and gp41); FV: HIV RNA^+^, p24^+/−^, antibody EIA^+^, WB^+^ (≥3 antibodies and ≥2 specific antibodies, without the p34 band); and FVI: HIV RNA^+^, p24^+/−^, antibody^+^, complete WB. All were treatment-naive participants at enrollment. The presence and timing of onset of symptoms consistent with PHI were determined by examination of medical records and patient interview.

### Ethical approval and informed consent

The study protocol was approved by the Paris Saint Louis Ethics Committee, and all patients gave their written informed consent.

### Guidelines followed statement

All methods were carried out in accordance with relevant guidelines and regulations.

### Clinical samples

Samples from various compartments (blood, semen, seminal fluid, and saliva) were obtained on the same day. All samples were processed and stored at −80 °C within 4 h of collection.

### Quantification of HIV DNA and RNA

HIV-1 RNA was quantified in plasma, seminal fluid, and saliva using the AmpliPrep/COBAS TaqMan HIV v.2 with a limit of quantification of 20, 100, and 60 copies/ml, respectively. Total cell-associated HIV-1 DNA was quantified in whole blood and non-spermatozoid cells as described elsewhere (detection threshold of three copies/PCR)^35^. Results for whole blood are reported as HIV-1 DNA copy number/10^6^ peripheral blood mononuclear cells (PBMCs), taking into account the white blood cell number and the blood formula. Results for non-spermatozoid cells are reported as HIV-1 DNA copy number/10^6^ cells.

### *env* V3 sequence analysis

HIV-1 RNA was extracted from plasma, seminal fluid, and saliva using the EasyMag (bioMérieux, Marcy l’Etoile, France) kit according to the manufacturer’s instructions. HIV-1 DNA was extracted from whole blood and non-spermatozoid cells using the QiaSymphony DSP DNA protocol « blood » (Qiagen, Courtaboeuf. France). The C2V3 *env* gene between positions 7008–7385 of the reference sequence HXB2 was amplified using the ANRS protocol (http://www.hivfrenchresistance.org/ANRS-procedures.pdf). Amplicons were multiplexed and used for UDS on a Roche/454 GS. Amplicons were quantified, fixed onto microbeads, subjected to emulsion PCR, and the beads loaded onto picotiter plates for forward and reverse pyrosequencing by means of the GS-FLX Titanium Kit in a Roche 4.5.4 GS Junior sequencer (454 Life Sciences, Roche Diagnostics Corp., Brandford, Connecticut). HIV 8E5 cells harboring one copy of HIV per genome were sequenced as a control to establish the error cut off.

### Bioinformatic analysis

#### Read filtering and de novo viral contigs

Demultiplexing was performed with the FASTX tool kit (http://hannonlab.cshl.edu/fastx_toolkit/), the adapters removed using Cutadapt^36^, and regions of low quality (phred score <20) removed using Trimmomatic^37^. Sequences with a minimum length of 40 bp were retained and used for the *de novo* assembly using Vicuna software^38^. *De novo* contigs were aligned using IndelFixer software (https://github.com/cbg-ethz/InDelFixer) to the respective reference according to the HIV subtype (HXB2 for subtype B and L39106.1 for CRF02_AG). The consensus sequences were obtained for each compartment using ConsensusFixer 0.4 software (https://github.com/cbg-ethz/ConsensusFixer).

#### Viral haplotype and phylogenetic analysis

The filtered reads were aligned to consensus sequences obtained in the last step using ngshmmalign software (https://github.com/cbg-ethz/ngshmmalign). The haplotypes were identified using the amplian.py script of the ShoRAH project^39^. Only haplotypes with a posterior probability > 95% were retained.

Multiple alignments of all the haplotypes of the same individual were built using mafft software^40^. The best sequence evolution model (lowest BIC) was identified using MEGA7^41^. This model was used as a parameter for MrBayes software in the phylogenetic tree identification^42^. Phylogenetic trees were represented with ggplot2 packages^43^ in R^44^.

#### Mean overall diversity

Reads of each of the compartments were aligned to reference sequences according to subtype^45^ using BWA software. The diversity was calculated using TN93 software (https://github.com/spond/TN93), which computes Tamura Nei pairwise distances between aligned sequences.

#### Generalized linear mixed model

The potential differential pressure between compartments was evaluated with a generalized linear mixed models. In these models the patients were the random effect while the compartments and the Fiebig stage were fixed effect predictors of diversity. In the first model, the analysis was limited to Fiebig stage IV and V patients. In the second model, the Fiebig stage IV and V patients were regrouped into a stage called “Later” and the only Fiebig stage II patient represents the “Early” stage. The random effects models were compared to a model of only fixed effects with the ANOVA test. The Markdown report in R is presented in the Supplementary Note S1.

#### Positive selection and N-glycosylation

Amino-acid changes under positive selection were identified in the multiple alignments of the haplotypes in each patient using the CorMut R package (load ≥2 & frequency of mutation > 0.01). Potential N-glycosylation sites were identified using N-Glycosite^46^, (https://www.hiv.lanl.gov/content/sequence/GLYCOSITE/glycosite.html).

#### Compartmentalization analysis

Viral compartmentalization was evaluated by computing using both distance-based (fixation index, Fst)^47^ and tree-based methods (Simmonds association index, Slatkin and Maddisson test, and correlation coefficient)^48^. The Fst was calculated using TN93 genetic distance (https://github.com/spond/TN93). Statistical significance was assessed via a 100-population permutation. We made clusters of reads using the cd-hit-est tool^49^ with the aim of reducing the potential influence of amplification errors. The representative sequence of each cluster was used to perform a second estimation of the Fst. The Slatkin and Maddison test was performed using Hyphy software^50^.

The p-values were estimated from 10,000 permutations of haplotypes between the compartments. P-values were corrected using Bonferroni correction. Populations among various compartments were defined as being compartmentalized if the three compartmentalization tests were significant (*P* < 0.0001).

## Supplementary information

Supplementary Information.
