# Supplementary material for: Characterization of HIV-1 diversity in various compartments at the time of primary infection by ultradeep sequencing
Source: Sci Rep. 2020 Feb 12;10:2409. doi: 10.1038/s41598-020-59234-6 (PMC7016127; doi:10.1038/s41598-020-59234-6)
Supplement: Supplementary file 1 — Supplementary Information. [file 41598_2020_59234_MOESM1_ESM.pdf]

Ultradeep sequencing characterization of HIV-1 diversity in different compartments in primary infected patients

Géraldine Gaube [1, +], Alix Armero [2,3,*, +], Maud Salmona [2,3], Marie-Laure Néré [2], Nadia Mahjoub [2], Caroline Lascoux-Combe [2], Audrey Gabassi [2], Sébastien Gallien [2], Jean Michel Molina [2,3], Constance Delaugerre [2,3] and Marie-Laure Chaix [2,3]

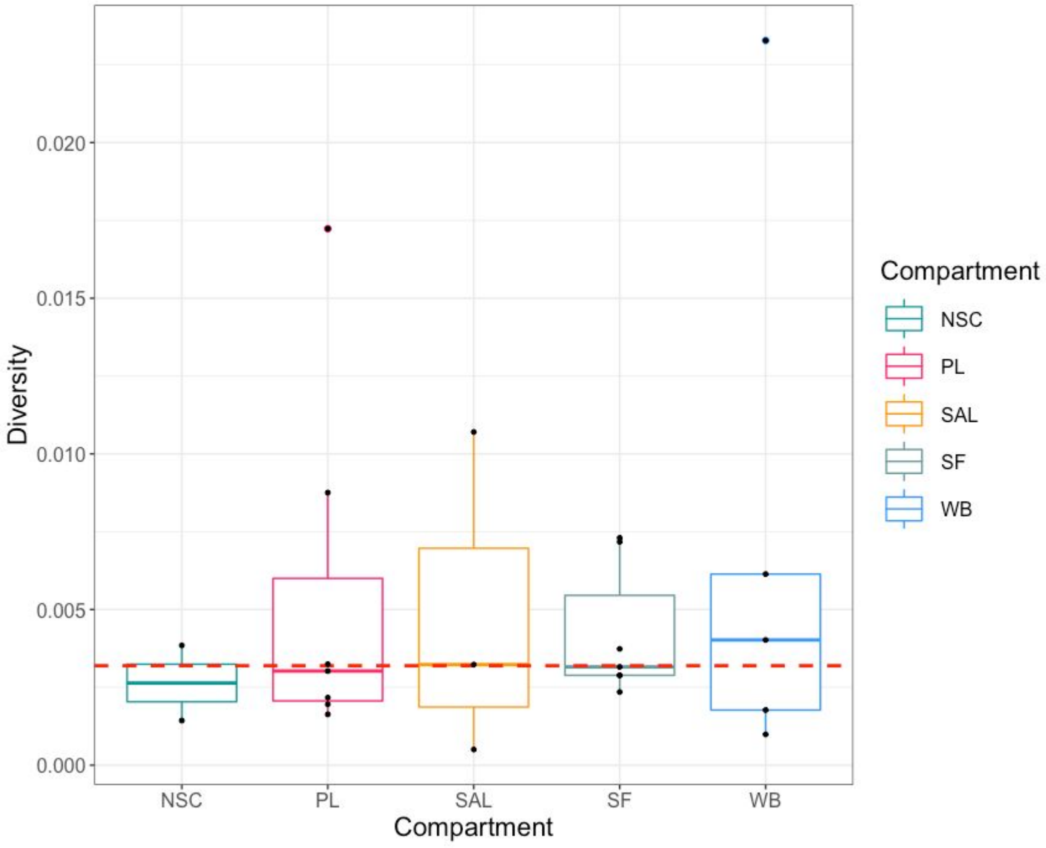

Supplementary Figure S1. Diversity by compartment of patients in the late Fiebig stages. Diversity is represented for Fiebig stage IV and V patients (black points). The dotted red line represents the median of the data pool. **SF**: seminal fluid, **WB**: Whole blood, **PL**: Plasma, **SAL**: saliva, **NSC**: non spermatozoid cells.

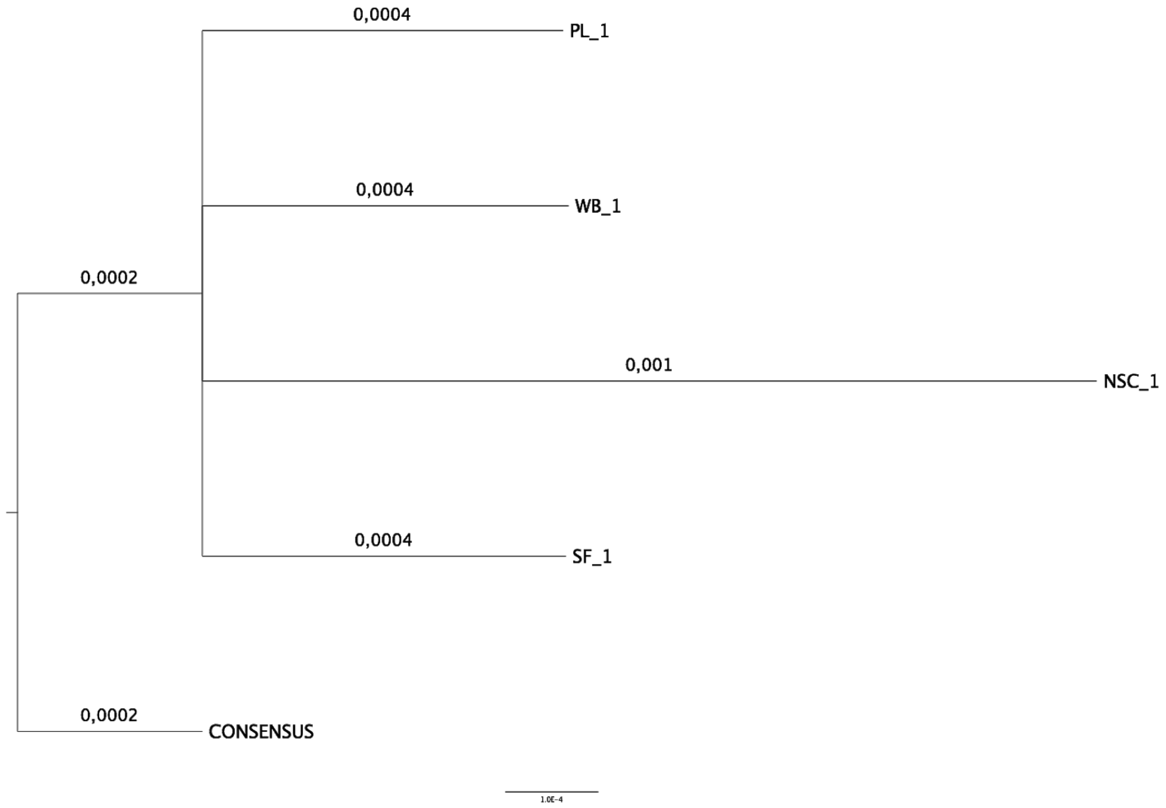

Supplementary Figure S2. Phylogenetic tree from a patient with low genetic diversity between HIV haplotypes. The poor diversity of HIV haplotypes leading to the characteristic start-like phylogeny in the P4 sample. In the branches are indicate the derived genetic distances. **SF**: seminal fluid, **WB**: Whole blood, **PL**: Plasma, **NSC**: non spermatozoid cells.

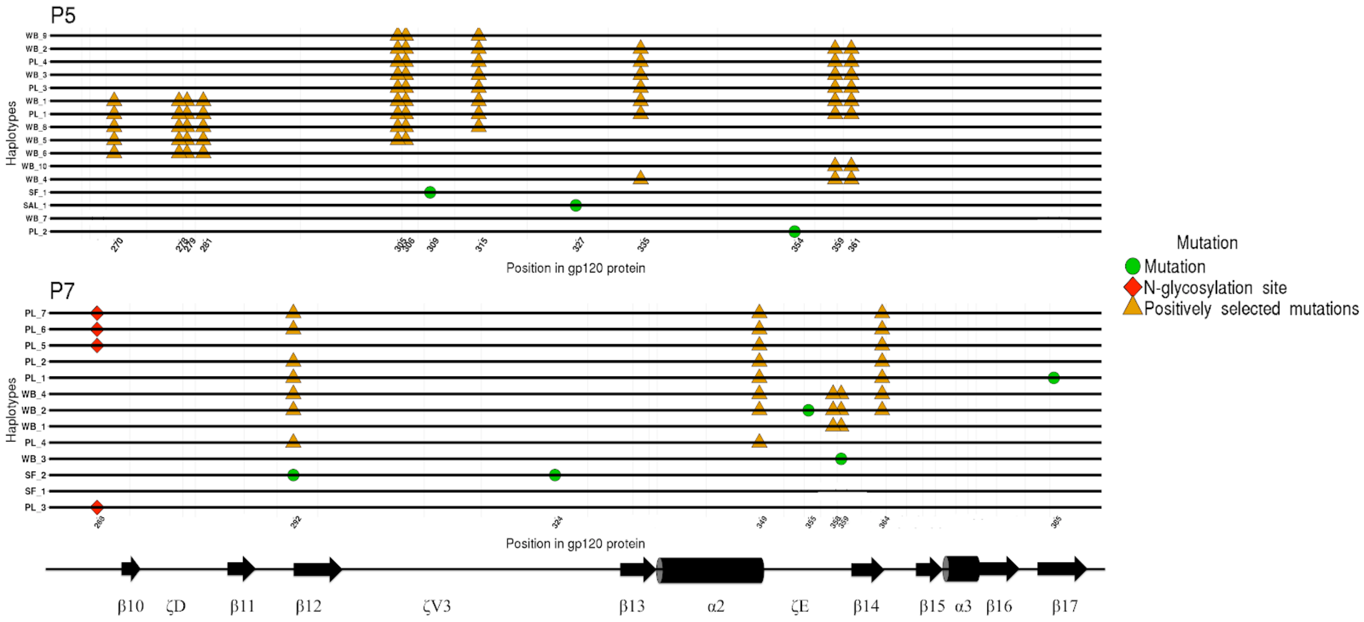

Supplementary Figure S3. Amino acid mutations under positive selection in HIV haplotypes of P5 and P7. The positions of all mutations observed in the haplotypes of P5 and P7 are presented according to the position in the gp120 protein. At the bottom of the figure the secondary structure of the gp120 protein is represented.

	Compartment	Number of HIV haplotypes
P2	SF	1
P2	WB	1
P2	PL	1
P3	WB	1
P3	PL	1
P3	SF	1
P4	SF	1
P4	NSC	1
P4	WB	1
P4	PL	1
P5	WB	10
P5	PL	4
P5	SF	1
P5	SAL	1
P6	SF	2
P6	SAL	1
P6	WB	1
P6	NSC	1
P6	PL	1
P7	WB	4
P7	PL	7
P7	SF	2
P8	PL	1
P8	SF	1
P8	SAL	2

Supplementary Table 1. Number of HIV haplotypes identified in different compartments of seven patients in early infection. **SF**: seminal fluid, **WB**: Whole blood, **PL**: Plasma, **SAL**: saliva, **NSC**: non spermatozoid cells

Supplementary Note S1. GLMMs HIV diversity in primary infection

Alix Armero

2019-11-27

Libraries

```
library(knitr)
library(ggplot2)
library(nlme)
```

Data: Mean overall diversity in the compartments of 8 patients with primary infection.

```
Div <- read.table("Diversity.csv")
colnames(Div)  <- c("Patient", "Fiebig", "Compartment", "Diversity")
```

Model 1. Compartment and Fiebig stage as fixed effects and Patient as random effect. Model without the Fiebig stage II patient.

```
Div_late <-  Div[Div$Fiebig!="II", ]
table(Div_late$Fiebig)
```

```
##
## II IV  V
##  0  6 18
```

```
model_late_rd <- lme(Diversity ~ Compartment+Fiebig, data = Div_late, random = ~ 1 | Patient)
summary(model_late_rd)
```

```
## Linear mixed-effects model fit by REML
##  Data: Div_late
##          AIC       BIC  logLik
##   -114.1332 -107.0102 65.0666
##
## Random effects:
##  Formula: ~1 | Patient
##         (Intercept)    Residual
## StdDev:  0.00386509 0.004288407
##
## Fixed effects: Diversity ~ Compartment + Fiebig
##                        Value   Std.Error DF    t-value p-value
## (Intercept)     0.0027863017 0.004695349 13  0.5934174  0.5631
## CompartmentPL   0.0009229121 0.003658119 13  0.2522914  0.8048
## CompartmentSAL -0.0008613614 0.004194070 13 -0.2053760  0.8405
## CompartmentSF  -0.0002948779 0.003658119 13 -0.0806091  0.9370
## CompartmentWB   0.0021829624 0.003757273 13  0.5809965  0.5712
## FiebigV         0.0024108866 0.003842887  5  0.6273634  0.5580
##  Correlation:
##                (Intr) CmprPL CmpSAL CmprSF CmprWB
## CompartmentPL  -0.673
## CompartmentSAL -0.598  0.696
## CompartmentSF  -0.673  0.804  0.696
## CompartmentWB  -0.626  0.760  0.655  0.760
## FiebigV        -0.648  0.081  0.095  0.081  0.057
##
```

```
## Standardized Within-Group Residuals:
##        Min         Q1        Med         Q3        Max
## -1.8680881 -0.3713151 -0.1430471  0.2964351  2.3090807
##
## Number of Observations: 24
## Number of Groups: 7
```

Fixed effects model

```r
model_late_fx <- gls(Diversity ~ Compartment+Fiebig, data = Div_late)
summary(model_late_fx)
```

```
## Generalized least squares fit by REML
##   Model: Diversity ~ Compartment + Fiebig
##   Data: Div_late
##        AIC       BIC   logLik
##   -112.101 -105.8684 63.05051
##
## Coefficients:
##                       Value   Std.Error    t-value p-value
## (Intercept)    -0.000145859 0.004885516 -0.0298554  0.9765
## CompartmentPL   0.003587078 0.004647421  0.7718427  0.4502
## CompartmentSAL  0.003101199 0.005295095  0.5856739  0.5654
## CompartmentSF   0.002369288 0.004647421  0.5098071  0.6164
## CompartmentWB   0.005156703 0.004811377  1.0717728  0.2980
## FiebigV         0.002786079 0.002747490  1.0140453  0.3240
##
##  Correlation:
##                (Intr) CmprPL CmpSAL CmprSF CmprWB
## CompartmentPL  -0.814
## CompartmentSAL -0.728  0.692
## CompartmentSF  -0.814  0.784  0.692
## CompartmentWB  -0.758  0.749  0.660  0.749
## FiebigV        -0.562  0.169  0.173  0.169  0.114
##
## Standardized residuals:
##        Min         Q1        Med         Q3        Max
## -1.1916939 -0.4891025 -0.1921134  0.2932739  2.7096786
##
## Residual standard error: 0.005713068
## Degrees of freedom: 24 total; 18 residual
```

ANOVA

```r
anova_late <- anova( model_late_fx, model_late_rd)
anova_late
```

```
##               Model df       AIC       BIC   logLik   Test L.Ratio p-value
## model_late_fx     1  7 -112.1010 -105.8684 63.05051
## model_late_rd     2  8 -114.1332 -107.0102 65.06660 1 vs 2 4.03217  0.0446
```

Model 2. Compartment and Fiebig stage as fixed effects and Patient as random effect. Fiebig stage IV and V make the "Late" group and the Fiebig stage II makes the "Early" group.

```r
Div_early_late <- Div
Div_early_late$Fiebig  <- as.character(Div_early_late$Fiebig)
Div_early_late$Fiebig[Div_early_late$Fiebig!="II"] <-  "Late"
Div_early_late$Fiebig[Div_early_late$Fiebig=="II"] <-  "Early"
```

```r
Div_early_late$Fiebig <- as.factor(Div_early_late$Fiebig)
table(Div_early_late$Fiebig)
```

```
##
## Early  Late
##     3    24
```

```r
model_early_late_rd <- lme(Diversity ~ Compartment+Fiebig, data = Div_early_late,
                           random = ~ 1 | Patient)
summary(model_early_late_rd)
```

```
## Linear mixed-effects model fit by REML
##  Data: Div_early_late
##         AIC       BIC   logLik
##   -139.5003 -131.1441 77.75014
##
## Random effects:
##  Formula: ~1 | Patient
##          (Intercept)    Residual
## StdDev: 0.003706995 0.004039397
##
## Fixed effects: Diversity ~ Compartment + Fiebig
##                           Value   Std.Error DF    t-value p-value
## (Intercept)      0.0025394098 0.005422027 15  0.4683506  0.6463
## CompartmentPL    0.0006478538 0.003404972 15  0.1902670  0.8517
## CompartmentSAL -0.0011175564 0.003934743 15 -0.2840227  0.7803
## CompartmentSF  -0.0001978499 0.003404972 15 -0.0581062  0.9544
## CompartmentWB    0.0017634967 0.003482524 15  0.5063847  0.6199
## FiebigLate       0.0021513801 0.004695512  6  0.4581779  0.6630
##  Correlation:
##                (Intr) CmprPL CmpSAL CmprSF CmprWB
## CompartmentPL  -0.551
## CompartmentSAL -0.435  0.699
## CompartmentSF  -0.551  0.824  0.699
## CompartmentWB  -0.545  0.791  0.666  0.791
## FiebigLate     -0.786  0.045 -0.005  0.045  0.064
##
## Standardized Within-Group Residuals:
##        Min         Q1        Med         Q3        Max
## -2.0318056 -0.3362031 -0.1840745  0.2455868  2.5307302
##
## Number of Observations: 27
## Number of Groups: 8
```

Fixed effects model

```r
model_early_late_fx <- gls(Diversity ~ Compartment+Fiebig, data = Div_early_late)
summary(model_early_late_fx)
```

```
## Generalized least squares fit by REML
##   Model: Diversity ~ Compartment + Fiebig
##   Data: Div_early_late
##         AIC       BIC  logLik
##   -136.4304 -129.1188 75.2152
##
## Coefficients:
##                       Value   Std.Error   t-value p-value
## (Intercept)     0.000319850 0.005156715 0.0620259  0.9511
## CompartmentPL   0.002719548 0.004349296 0.6252846  0.5385
```

```
## CompartmentSAL 0.002172506 0.004998008 0.4346743  0.6682
## CompartmentSF  0.001873844 0.004349296 0.4308384  0.6710
## CompartmentWB  0.004278789 0.004506262 0.9495208  0.3532
## FiebigLate     0.002320370 0.003406414 0.6811769  0.5032
##
##  Correlation:
##                (Intr) CmprPL CmpSAL CmprSF CmprWB
## CompartmentPL  -0.733
## CompartmentSAL -0.582  0.689
## CompartmentSF  -0.733  0.802  0.689
## CompartmentWB  -0.728  0.777  0.665  0.777
## FiebigLate     -0.661  0.098  0.000  0.098  0.126
##
## Standardized residuals:
##        Min         Q1        Med         Q3        Max
## -1.0831537 -0.4777429 -0.2888591  0.3024082  2.9878283
##
## Residual standard error: 0.005475044
## Degrees of freedom: 27 total; 21 residual
```

ANOVA

```
anova_early_late <- anova(model_early_late_fx, model_early_late_rd )
anova_early_late
```

```
##                     Model df       AIC       BIC   logLik   Test  L.Ratio
## model_early_late_fx     1  7 -136.4304 -129.1188 75.21520
## model_early_late_rd     2  8 -139.5003 -131.1441 77.75014 1 vs 2 5.069867
##                     p-value
## model_early_late_fx
## model_early_late_rd  0.0243
```
